# Biotechnological synthesis of Pd-based nanoparticle catalysts

**DOI:** 10.1039/d1na00686j

**Published:** 2021-12-21

**Authors:** Christopher Egan-Morriss, Richard L. Kimber, Nigel A. Powell, Jonathan R. Lloyd

**Affiliations:** Department of Earth and Environmental Sciences, Williamson Research Centre for Molecular Environmental Science, University of Manchester UK jon.lloyd@manchester.ac.uk; Department of Environmental Geosciences, Centre for Microbiology and Environmental Systems Science, University of Vienna 1090 Vienna Austria; Johnson Matthey Technology Centre Reading RG4 9NH UK

## Abstract

Palladium metal nanoparticles are excellent catalysts used industrially for reactions such as hydrogenation and Heck and Suzuki C–C coupling reactions. However, the global demand for Pd far exceeds global supply, therefore the sustainable use and recycling of Pd is vital. Conventional chemical synthesis routes of Pd metal nanoparticles do not meet sustainability targets due to the use of toxic chemicals, such as organic solvents and capping agents. Microbes are capable of bioreducing soluble high oxidation state metal ions to form metal nanoparticles at ambient temperature and pressure, without the need for toxic chemicals. Microbes can also reduce metal from waste solutions, revalorising these waste streams and allowing the reuse of precious metals. Pd nanoparticles supported on microbial cells (bio-Pd) can catalyse a wide array of reactions, even outperforming commercial heterogeneous Pd catalysts in several studies. However, to be considered a viable commercial option, the intrinsic activity and selectivity of bio-Pd must be enhanced. Many types of microorganisms can produce bio-Pd, although most studies so far have been performed using bacteria, with metal reduction mediated by hydrogenase or formate dehydrogenase enzymes. Dissimilatory metal-reducing bacteria (DMRB) possess additional enzymes adapted for extracellular electron transport that potentially offer greater control over the properties of the nanoparticles produced. A recent and important addition to the field are bio-bimetallic nanoparticles, which significantly enhance the catalytic properties of bio-Pd. In addition, systems biology can integrate bio-Pd into biocatalytic processes, and processing techniques may enhance the catalytic properties further, such as incorporating additional functional nanomaterials. This review aims to highlight aspects of enzymatic metal reduction processes that can be bioengineered to control the size, shape, and cellular location of bio-Pd in order to optimise its catalytic properties.

## Abstract 2

Cell supported Pd metal nanoparticles are sustainably synthesised *via* microbial bioreduction. Bio-Pd nanoparticles are effective heterogeneous catalysts that catalyse industrially important reactions, such as hydrogenation and C–C coupling reactions.

## Graphical abstract

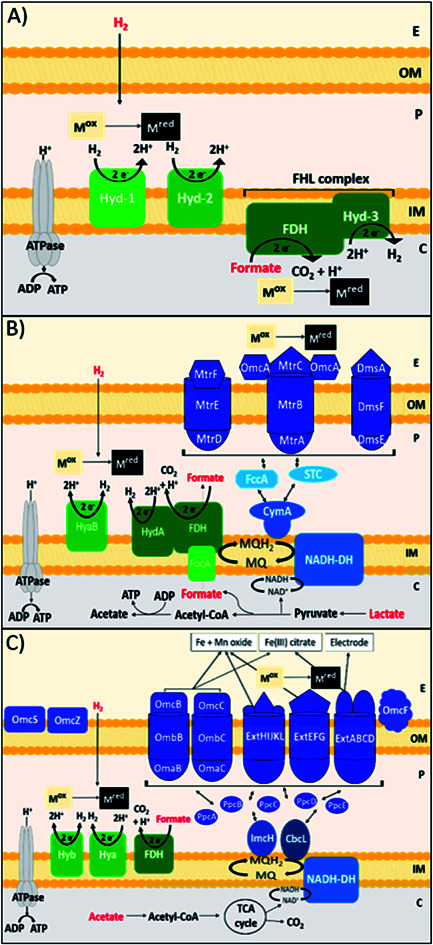

## 1. Introduction

Catalysts are vital in modern industry; they are responsible for 30% of Europe's gross domestic product, and are required in the processing of 80% of all manufactured products.^1^ Supported palladium nanoparticles are important catalysts that can catalyse a wide array of industrially important reactions, such as hydrogenation, hydrogenolysis, dehydrogenation, and C–C couplings such as Heck, Suzuki, and Sonogashira reactions.^2–4^ These reactions are used to synthesise fine chemicals, which are essential feed stocks for the pharmaceutical, agrochemical and lifestyle product industries. Some reactions can only be performed with a catalyst, and in many cases catalysts offer shortcuts in chemical synthesis routes that replace a significant number of stoichiometric steps. A vast amount of waste is produced from stoichiometric reactions, for example, a kilogram of product can also produce up to 100 kg of by products such as inorganic salts, metal hydrides and mineral acids.^5^ Palladium catalysis also has drawbacks however, such as the high cost of the metal and the need to avoid catalyst poisoning by sulfur, which can inhibit reactant surface adsorption at low degrees of surface coverage.^6^ Furthermore, the demand for palladium far outstrips the global supply, leading to high prices. Pd has applications as a catalyst in autocatalytic converters, and in the chemicals industry, as well as having uses in electronics, dental implants, and jewellery.^7^ Therefore technologies that enable the sustainable reuse of Pd are of utmost importance. In order to reduce waste and increase the sustainability of fine chemical production, there is an ongoing need for new catalysts with higher catalytic activity, better selectivity, longer life time, and minimal loss in activity with ageing.^8,9^

Catalysts are broadly separated into two classes, homogeneous catalysts that exist in the same phase as the reactants they are catalysing and heterogeneous catalysts that exist in a separate phase. Homogeneous Pd catalysts typically consist of soluble Pd complexes stabilised in solution by a variety of ligands.^10^ Heterogeneous Pd catalysts are typically nanoparticles stabilised on a support material such as activated carbon, zeolites, metal oxides, earth metal salts, or polymers.^11,12^ Homogeneous Pd catalysts are often preferred for industrial applications, in part due to the stereoselectivity they provide, which cannot be obtained with heterogeneous catalysts. However, the ligands required whilst popular and efficient, are also toxic and/or expensive.^13^ Homogeneous catalysts can be difficult to separate from a reaction mixture, making their reuse or recycling challenging. The metal or ligand may end up in the final product, such as in pharmaceuticals, and must be removed.^14^ Heterogeneous catalysts are more sustainable; they can be separated more easily from a reaction system by simple filtration, enabling them to be reused several times, and in the absence of leaching, the product is not contaminated with transition metals or ligands.^15^ Supported Pd catalysts make up around 30% of the fine chemicals sector, and are most commonly used for hydrogenation reactions.^16^ However, supported Pd nanoparticles have limitations including lower (or absent) stereoselectivity and lower activity. This may be compensated for with higher reaction temperatures, but thermal degradation of the substrates or products may occur.^17^

Palladium metal nanoparticles produced by microorganisms (bio-Pd), under environmentally benign conditions, offer a potentially sustainable solution to the issues highlighted above. Bio-Pd is synthesised *via* enzymatic reduction at ambient temperature and pressure, simply requiring inexpensive buffer solutions and an electron donor, such as organic acids or hydrogen.^18,19^ The formation of metallic nanoparticles *via* enzymatic reduction from industrial waste solutions has been demonstrated, potentially revalorising these waste streams and allowing the recovery and reuse of precious metals.^20,21^ Microbial cells contain proteins and other biomolecules with high affinities for metals that help to prevent agglomeration, and control the size of nanoparticles without the need for toxic and expensive chemicals, such as chemical capping agents.^22,23^ Bio-Pd can also be reused several times whilst retaining catalytic activity and selectivity.^24–26^

Bacteria were first shown to bind cationic metal species *via* electrostatic interaction to their anionic cell surface in the common Gram-positive soil bacterium *Bacillus subtilis*.^27^ After binding metals to their surface, many different species of microbe are capable of reducing the metal ions to produce metal nanoparticles, for example from radionuclides, and transition metals.^28^ A two-step mechanism was proposed for the bioreduction of metals *via* bacterial cells; first the metal ion adsorbs to an active site on the cell surface, which then acts as a nucleation site for the reduction of metal ions from solution and their subsequent growth.^29^ Microbes generate the electrons for bioreduction from the enzymatic oxidation of an electron donor, and these electrons are transferred through cellular enzymatic pathways to a terminal reductase, where the metal species act as an electron acceptor.

Lloyd *et al.* first reported the bioreduction of soluble Pd(ii) to form Pd(0) nanoparticles in the Gram-negative sulfate-reducing bacterium *Desulfovibrio desulfuricans*.^18^ Bio-Pd has since been produced by many different microbes, and its catalytic properties widely explored. Bio-Pd has performed comparably to commercial supported Pd catalysts, such as Pd/C, for a range of reactions, including hydrogenation reactions, C–C coupling reactions, and remediative reactions such as the reduction of Cr(vi) to Cr(iii), or the dehalogenation of environmental pollutants.^26,30–32^ Recently, with the addition of precious and noble metals, microbially produced bimetallic nanoparticles have also been produced that possess improved catalytic properties over bio-Pd.^33–36^

Microbially supported metal nanoparticles are promising green catalysts, however, in order to be commercially viable they must be bioengineered to have higher catalytic activity and/or selectivity, be more durable, less expensive at scale, or catalyse a wider range of reactions than current commercially available catalysts.^37^ This review will aim to highlight the research into bio-Pd synthesis required to achieve these goals, and assess the catalytic properties achieved so far from microbial bionanocatalyst systems.

## 2. Design of supported palladium catalysts

### 2.1 Size and shape of nanoparticles

The electron configuration of Pd makes it a very stable catalyst, as reactants are able to bind to the metal surface and form intermediate states that can react to form the final product. Pd nanoparticles are excellent catalysts as they have a high proportion of highly reactive surface atoms. The particle size determines the ratio of surface-to-bulk metal atoms, with most industrial catalysts requiring metal nanoparticles in the 1–20 nm range to provide high enough specific surface areas.^38^ On the other hand, the particle shape is related to the types of facets on the particle surface, which affects their reactivity.^39^ Atoms with lower coordination numbers, *i.e.* fewer bonded atoms, are generally more reactive, and surface atoms tend to have a decreasing coordination number in the order of face > edge > vertex. For example, in fcc metals such as Pd, {111} facets have a coordination number of 9, {110} and {100} facets have coordination numbers of 8 and 7, and defects such as steps or kinks can have a coordination number of 6.^40^ Nanoparticles can catalyse reactions that generate multiple potential products. The selectivity towards a certain product is determined by reactants binding to the metal surface in a specific configuration that allows a certain atom in a molecule to react.

Pd catalysed selective hydrogenations are important industrial reactions, for example converting alkynes to alkenes for the synthesis of polymers. Rosenmund, and later Lindlar, developed Pd catalysts poisoned with barium, lead or sulfur to lower their catalytic activity and increase their selectivity.^41,42^ Modern catalysts avoid using these toxic metals, instead focusing on the effects of particle size, the role of facets and atomic surface sites, and using modifiers to specify stereochemistry and enhance selectivity.^43–45^ For example, the hydrogenation of benzene on a Pt{111} single-crystal surface gave a mix of cyclohexane and cyclohexene, while only cyclohexene was generated on Pt{100}.^46^ Larger Pd nanoparticles (18 nm) and plane surface atoms dominated by {100} and {111} facets were shown to favour semi-hydrogenation; whereas smaller nanoparticles (6 nm) containing edge and corner sites favoured full hydrogenation.^47^ The hydrogenation of pentene was shown to experience a strong particle size effect, whereas ethene hydrogenation was structure insensitive.^48^ Therefore, although decreasing the particle size increases activity, selectivity may not be favourable, and controlling the size and monodispersity of bio-Pd, along with the shape and surface facets available, would be highly desirable to tailor its catalytic properties. Although some control over size has been established, fairly little characterisation of the shape of bio-Pd nanoparticles has yet been reported.^26,49^

The abiotic formation of nanoparticles from solution is generally divided into two steps; nucleation of seed particles, and their subsequent growth. The crystal structure of seed particles often controls the shape of nanoparticles, along with the capping agents used and the rate of atomic deposition.^39^ The size of nanoparticles depends on their growth and is often controlled by the type and concentration of capping agent and solvent, and the reduction rate.^50^ For example, shape and size control of Ag nanoparticles was achieved by altering the ratio between the growth rates of {100} and {111} planes using different seeds and types of capping agents.^22^ In terms of bio-Pd, the rate of metal bioreduction will depend on the type of organism and electron donor used. The Pd(ii) complexes that adsorb to the cell in the initial biosorption step may act as seeds that contribute to the final size and shape of the nanoparticles. Cell biomolecules contain functional groups that bind metals,^51^ and peptides have been shown to act as capping agents that may direct or inhibit growth of nanoparticles, controlling their size and function.^52,53^ In addition, these biomolecules will be responsible for stabilising bio-Pd nanoparticles during multiple reaction cycles, as the adsorption of reaction species during catalytic operation can alter nanoparticle size. For example the average Pd crystallite size was shown to increase from 2.4 nm to 23 nm in 5% Pd/C, after use in the Heck reaction.^54^

### 2.2 Support material

The catalytic effect of supported metal nanoparticles is mainly governed by the chemical properties of the active metal phase. The support distributes the active phase over a large surface area, however, further interactions involving the support material must not be overlooked.^55^ Studies of chemically synthesised metal nanoparticles show that the support material can be critical to the efficiency and stability of the catalytic system, affecting the appearance of specific active sites at the metal–support boundary, variations in metal nanoparticle shape and crystal structure, and changes in the metal particle charging effects.^56^ Cooperative catalysis can occur between functional groups present on the support material that operate alongside the catalytic metal surface during a chemical reaction, assisting in the adsorption and desorption of reactants.^57^ For example, a hydrophilic carbon support enabled high selectivity for the hydrogenation of phenol to cyclohexanone by Pd nanoparticles.^58,59^ In contrast removing oxygen surface groups from various carbon materials assisted the selective hydrogenation of cinnamaldehyde towards cinnamyl alcohol for various platinum group metal (PGM) catalysts.^60,61^ The biomolecules within the cellular envelope that bind and stabilise bio-Pd nanoparticles contain a high density of functional groups, which may play a role in a given reaction. For example, in an early study by De Windt *et al.* the dechlorination of the hydrophobic polychlorinated biphenyl (PCB) 2,3,4-trichlorobiphenyl was most effectively catalysed by bio-Pd when large Pd(0) nanoparticles covered most of the hydrophilic cell surface.^30^ However, when nanoparticles up to 100-fold smaller were present, embedded within the hydrophilic cell surface, the PCB was not significantly degraded, possibly due to the lack of binding sites at the cell surface. Conversely, the opposite relationship was observed for the dechlorination of the anionic contaminant perchlorate, which presumably would find favourable binding sites on protonated cell surface functional groups.

Other important aspects of heterogeneous catalysts are their recovery and reusability. The support material must allow separation from the reaction mixture; for carbon supports this means high attrition resistance in order to avoid generating extremely fine particles that make filtration.^16^ Bio-Pd can be simply filtered from reaction mixtures and reused, and may be more resistant to Pd nanoparticle aggregation on reuse than Pd/C.^26^ The cell support material is amenable to various processing techniques that could alter the cell surface chemistry, enhancing the support's role in certain reactions, and making the bionanoparticle more reusable (see Section 8).

## 3. Pd biosorption by microbes

Microbes are excellent biosorbents of metal species due to their high surface-to-volume ratios and high contents of potentially active chemisorption sites.^62^ The biosorption of precious metals by microbes, or biomaterials derived from them, has been of great interest to recover metals from waste solutions.^63–65^ Biosorption is the first step in the bioreduction process, and likely contributes to the size, shape and localisation of bio-Pd nanoparticles. Biosorption is the passive sequestration by biomass of metal species from solution; this is an abiotic process that occurs on cells whether live or dead.^63^ Bioaccumulation can follow in some examples, and is the metabolically active transport of metal species into the cell. Bioreduction of metal species to a potentially less mobile state may occur in the cytoplasm, at the cell surface, or extracellularly *via* secretion of metabolites.^66^ Pd(ii) bioreduction also occurs abiotically such as in the presence of H_2_ or formate. Microbial cell surfaces contain a wide array of biomolecules such as chitin (in fungi), and peptidoglycan and lipopolysaccharide (in bacteria), which in turn contain many different functional groups that can bind metals.^67,68^ In addition, PGMs in solution can form an array of anionic, neutral, and cationic complexes depending on the pH, ligands, and ions in solution, all of which in turn affects the cell surface chemistry. Biosorption can therefore occur through different mechanisms, including ionic interactions such as ion exchange, electrostatic forces, hydrogen bonding or van der Waals forces, or through covalent interactions such as coordination.^66^

Although the mechanism of Pd(ii) biosorption can vary across microbial species, Pd(ii) biosorption has been reported to occur at amine, carboxyl, hydroxyl and phosphoryl groups.^69–75^ The N and O atoms of these groups can act as ligands that coordinate to Pd(ii). These functional groups can also become protonated, or deprotonated, with changing pH, which changes their ionic interactions with Pd(ii) complexes. Many microbes display strongly pH-dependent Pd(ii) biosorption due to ionic interactions. In the yeast *Saccharomyces* sp. optimal Pd(ii) biosorption occurred at pH = 2–3, due to electrostatic interactions between anionic [PdCl_4_]^2−^ and [PdCl_3_(H_2_O)]^−^ complexes and protonated functional groups.^76^ However, the biosorption capacity was much lower outside this narrow pH range. At strongly acidic pH (pH < 2) biosorption decreased due to Cl^−^ ions competing with [PdCl_4_]^2−^ for protonated cell surface sites. At pH >3.5, hydrolysis of Pd(ii) complexes leads to different aquachloro- (*e.g.* [PdCl_3_(H_2_O)]^−^) and chlorohydroxo- (*e.g.* [PdCl_3_(OH)]^2−^) complexes forming, which may have low affinity for the metal binding functional groups available. Similar biosorption behaviour was also observed for the alga *Chlorella vulgaris*, with optimal biosorption at pH = 1–2.^77^ On the other hand, in the fungus *Aspergillus* sp., optimal Pd(ii) biosorption was recorded at pH = 4–11.^76^ Given that Pd(ii) biosorption was mostly pH-independent, Pd(ii) complexes likely formed covalent interactions, such as complexation, with the various functional groups in the fungal cell wall. The amine groups of chitosan, chitin and glucan, and amine groups more generally, have been shown to effectively complex Pd(ii).^68,78–81^

Yong *et al.* showed that the choice of Pd(ii) precursor impacted biosorption by *D. desulfuricans*. Biosorption of [PdCl_4_]^2−^ was pH-dependent from pH = 1–7, suggesting an ionic biosorption mechanism, with optimal biosorption occurring at pH = 4. On the other hand, the biosorption of [Pd(NH_3_)_4_]^2+^ was pH-independent, maintaining around 20% biosorption across the measured pH = 1–7, suggesting a covalent mechanism.^82^ The pH would affect the cell surface charge in both cases. However, whereas pH would have a significant effect on the speciation of the aquachloropalladate complex formed, the tetraamine complex would be largely unaffected by pH. Bioreduction of [Pd(NH_3_)_4_]^2+^ was optimal at pH = 7, and the rate of Pd(ii) reduction fell as the pH decreased.^82^ The reduction rate decreased faster at pH < 7 with formate as electron donor compared to H_2_, because H_2_ is a stronger abiotic reducing agent and formate driven reduction relies more on the cell's enzymatic bioreduction.

In an impressive study by Deplanche *et al.*, seven different species of bacteria were assessed for their Pd(ii) biosorption capacity, bioreduction, and the subsequent activity of the resulting bio-Pd in catalytic reactions.^83^ The biosorption capacity was strongly correlated to the bioreduction rate, and Gram-negative species possessed far superior Pd(ii) biosorption capacity over the two Gram-positive species at the acidic pH (pH = 2.3) used in this study. Anionic Pd complexes would have been the dominant Pd(ii) species under these conditions, which would be electrostatically attracted to protonated functional groups. Pd(ii) reportedly coordinates to the carboxyl groups of the S-layer of Gram-positive bacteria, which may have been deteriorated significantly by the acidic pH,^70^ whereas coordination to the carboxyl and amine groups of peptidoglycan in the Gram-negative bacteria was less affected.^83^ Several studies have demonstrated that competing ions in solution can significantly decrease Pd(ii) biosorption, and consequently bioreduction. [PdCl_4_]^2−^ biosorption by *D. desulfuricans* was significantly lower in the presence of Cl^−^ ions than NO_3_^−^ or SO_4_^2−^ ions, from their respective acids at pH = 1–4.^84^ Increasing the Cl^−^ concentration at pH = 2 roughly halved the biosorption capacity and significantly reduced the bioreduction rate; whereas increasing NO_3_^−^ concentration had no effect on biosorption capacity or bioreduction rate.^84,85^ The effect of competing ions on biosorption of [Pd(NH_3_)_4_]^2+^ was not reported. However, the bioreduction rate of the ammonia complex was significantly reduced by increasing NO_3_^−^ concentration, and was completely inhibited at 100 mM of Cl^−^.^85^ The solution chemistry therefore has a significant impact on Pd(ii) biosorption and bioreduction; as has been shown to be the case for the bioreduction of other metals. For example, using different buffers drastically affected the products of Tc(vii) bioreduction by *S. oneidensis*.^86^

## 4. Bio-Pd from microbial bioreduction

Metal bioreduction is performed by many different microorganisms on a broad range of metal species, and contributes significantly to the biogeochemical cycling of trace elements. Metal-reducing bacteria couple the oxidation of an electron donor to the reduction of oxidized metal species, transferring electrons from an organic or inorganic substrate to the metal. In many examples, these processes support anaerobic metabolism and growth. Hydrogen is a ubiquitous electron donor in these bacteria, which use hydrogenase enzymes to catalyse the oxidation of H_2_ to form protons and electrons. Different groups of hydrogenases are classified by the metal atoms associated with their active centres, for example [NiFe]-hydrogenases have been identified to perform metal reduction.^87^ Formate is also a common electron donor, with formate dehydrogenase (FDH) enzymes used to catalyse the oxidation of formate to CO_2_ and H^+^. These enzymes can reduce metals directly, or in tandem, for example forming a formate hydrogenlyase (FHL) complex, as found in *E. coli*.^88^ An important development in the field has been the discovery and application of specialist dissimilatory metal-reducing bacteria (DMRB). In addition to possessing hydrogenase and FDH enzymes, DMRB are adapted to utilise a wider range of organic substrates as electron donors for metal reduction, and possess complex protein pathways to transport electrons from reactions in the cytoplasm to the outer cell surface.^89^

### 4.1 Bio-Pd from H_2_/formate oxidation

The bioreduction of Pd(ii) to Pd(0) was first studied in the Gram-negative sulfate-reducing bacterium (SRB) *Desulfovibrio desulfuricans*, using hydrogen as the electron.^18^ Pd(0) particles were localised to the periplasm, the location of the [NiFe]-hydrogenase in this bacterium. Many other species of bacteria have since been shown to reduce Pd(ii) using either formate or H_2_ as the electron donor (Table 1).

**Table 1:** Overview of bio-Pd systems catalysing various reactions, including the: species of bacteria, electron donor used for bioreduction, enzymatic reduction pathway, and the reaction catalysed in the study

Bacteria	Electron donor	Proposed reduction pathway	Catalytic application	Reference
*Arthrobacter oxidans*	H_2_	Hydrogenase + abiotic	Cr(vi) reduction	83
*Bacillus sphaericus*	H_2_	Hydrogenase + abiotic	Cr(vi) reduction	126
*Cupriavidus metallidurans*	H_2_	Hydrogenase + abiotic	Cr(vi) reduction	83
*Cupriavidus necator*	Formate	FDH + hydrogenase + abiotic	H_2_ from hypophosphite	97
*Desulfovibrio desulfuricans*	H_2_	Hydrogenase + abiotic	Cr(vi) reduction	83, 94, 126 and 127
*Desulfovibrio vulgaris*	H_2_	Hydrogenase + abiotic	Cr(vi) reduction	127
*Enterococcus faecalis*	Formate	FDH + hydrogenase + abiotic	Cr(vi) reduction	128
*Escherichia coli* (hydrogenase mutants)	H_2_	Hydrogenase + abiotic	Cr(vi) reduction	91
*Escherichia coli*	H_2_	Hydrogenase + abiotic	Cr(vi) reduction	83
*Geobacter sulfurreducens*	Formate	FDH + hydrogenase + abiotic	Cr(vi) reduction	129
*Klebsiella oxytoca*	Glucose	N/A	Azo dye reduction	130
*Micrococcus luteus*	H_2_	Hydrogenase + abiotic	Cr(vi) reduction	83
*Paracoccus denitrificans*	Formate	FDH + hydrogenase + abiotic	H_2_ from hypophosphite	97
*Pseudomonas putida*	Formate	FDH + hydrogenase + abiotic	H_2_ from hypophosphite	97
*Serratia* sp.	H_2_	Hydrogenase + abiotic	Cr(vi) reduction	83
*Shewanella loihica* PV-4	Lactate	Omc (MtrCAB)	Azo dye reduction	131
*Shewanella loihica* PV-4	Formate	FDH + hydrogenase + abiotic	Cr(vi) reduction	120
*Shewanella oneidensis*	Formate	FDH + hydrogenase + abiotic	4-Nitrophenol reduction	81
*Shewanella oneidensis*	Formate	FDH + hydrogenase + abiotic	Perchlorate reduction	30
*Shewanella oneidensis*	H_2_	Hydrogenase + abiotic	Cr(vi) reduction	83
*Shewanella oneidensis*	Formate	FDH + hydrogenase + abiotic	Reduction of nitrobenzene & 4-chlorophenol	96
*Shewanella oneidensis*	Lactate	Omc (MtrCAB)	4-Nitrophenol reduction	119
*Shewanella* sp. CNZ-1	Lactate	N/A	4-Nitrophenol reduction	119

Studies on gene knockout mutants have established the role of hydrogenases as a terminal reductase of Pd(ii) in bacterial model systems. In the Gram-negative SRB *D. fructosivorans*, a wild-type strain was compared to a periplasmic [NiFe]-hydrogenase deletion mutant, a periplasmic [Fe]-hydrogenase deletion mutant, and a double deletion mutant missing both hydrogenases.^90^ All of the mutants reduced Pd(ii) at a comparable rate to the wild type and deposited Pd(0) in the periplasm, with the exception of the [NiFe]-mutant, which deposited Pd(0) on the cytoplasmic side of the inner membrane, the location of the remaining hydrogenases. Pd was therefore shown to be deposited at the periplasmic site of the [NiFe]-hydrogenase enzyme, implicating it as the terminal reductase in Pd(ii) bioreduction.^90^ Similarly, Pd(0) deposition was found to occur at the site of hydrogenases in knockout studies of *E. coli*, when using formate as the electron donor.^91^*E. coli* contains at least three [NiFe]-hydrogenases; Hyd-1 and Hyd-2 are periplasmic inner membrane-bound enzymes, whereas Hyd-3 is associated with a cytoplasmic FHL complex on the inner membrane.^88^ When only Hyd-3 was present bio-Pd was mostly located on the cytoplasmic side of the inner membrane. When double deletion mutants left only periplasmic Hyd-1 active in the reduction of Pd(ii), bio-Pd was localised to, and spanned the width of, the periplasm. When only Hyd-2 was present bio-Pd was associated with the inner membrane. The mutant lacking all three hydrogenases did not appear to enzymatically reduce Pd(ii), producing large extracellular Pd deposits that were likely due to abiotic reduction by formate.^91^ The parent strain and the mutant containing Hyd-1 both produced a 5% Pd/*E. coli* (wt/dry wt) catalyst with comparable activity to a 5% Pd/C commercial catalyst for the reduction of Cr(vi). In comparison the mutants without Hyd-1 produced bio-Pd with lower catalytic activity, potentially due to the bio-Pd being less accessible at the inner membrane.^91^

The choice of electron donor in bioreduction leads to bio-Pd with different nanostructures and catalytic properties. Three different SRBs produced bio-Pd with superior activity for the reductive dehalogenation of chlorinated aromatic compounds, when supplied with formate as electron donor over hydrogen.^92^ Intracellular bio-Pd nanoparticles were mainly crystalline icosahedrons enclosed by {111} facets when formate was supplied as electron donor. When hydrogen was supplied the intracellular particles were amorphous.^49^ Formate driven bio-Pd in *E. coli* was reported to be smaller and more dispersed than hydrogen-driven bio-Pd.^93^ A key reason for these differences, as discussed above, are that hydrogen and formate are metabolised differently by cells, and result in different Pd(ii) bioreduction rates. The ability to control the size of bio-Pd nanoparticles is a necessary component in tailoring their catalytic properties.

One method for controlling the particle size of bio-Pd is to vary the concentrations of cells relative to the initial concentration of Pd(ii); typically expressed as the cell dry weight (CDW) to Pd(ii) ratio, or Pd loading of the cell as a weight percentage (wt/wt). Yong *et al.* demonstrated that increasing the concentration of biomass, or decreasing the initial Pd(ii) concentration, increased the relative amount of Pd(ii) biosorption to the cell.^82^ Mabbett and Mackaskie demonstrated that this metal loading affected the catalytic properties of bio-Pd. Pd/*D. desulfuricans* prepared with H_2_ as the electron donor at a 6 : 1 CDW : Pd ratio outperformed the same catalyst prepared at a 1 : 1 CDW : Pd ratio for the reduction of Cr(vi), reducing 90% and 70%, respectively, after 2 hours.^94^ The superior catalytic activity was attributed to smaller particle size, however Pd/*D. desulfuricans* prepared at a 10 : 1 ratio displayed negligible Cr(vi) reduction. The total metal loading impacts on the nanoparticle dimensions, and cellular location. For example, Attard *et al.* noted that at a 20 wt% loading Pt/*D. desulfuricans* nanoparticles were mostly observed at the cell surface with a mean size of 4.5 nm. In contrast, at a 1 wt% loading Pt/*D. desulfuricans* nanoparticles were embedded further within the cell envelope, with a smaller mean size of 2.3 nm.^95^ Hou *et al.* also showed that the CDW : Pd ratio affected the extracellular distribution of bio-Pd, which in turn impacted the catalytic activity of bio-Pd nanoparticles.^96^ Pd/*S. oneidensis* nanoparticles were synthesised using formate as the electron donor at five different CDW : Pd ratios: 6 : 1, 3 : 1, 1 : 1, 1 : 3 and 1 : 6, which increased the average particle size from <10 nm to >50 nm. When tested as a suspension catalyst for the reduction of nitrobenzene and 4-chlorophenol, the smallest nanoparticles gave some of the lowest rates of reaction. The FDH and hydrogenase enzymes that reduce Pd(ii) are periplasmic in *S. oneidensis*, therefore the smallest nanoparticles (<10 nm) synthesised at 6 : 1 CDW : Pd ratio were embedded in the periplasm and had the lowest cell surface coverage, exposed surface area, and extracellular distribution. The Pd nanoparticles synthesised at the 1 : 3 CDW : Pd ratio gave the highest rates of reaction. These particles possessed a larger average size of 25.8 nm, but with high exposed surface area, extracellular distribution, and cell surface coverage, indicating that these properties were more favourable than size.^96^ At lower CDW : Pd ratios there were fewer cell nucleation sites and the cells were exposed to more metal toxicity. The particles were larger and more often extracellular, as they nucleated in the periplasm then grew beyond the cell surface; in addition damaged cells would release enzymes providing additional extracellular nucleation sites, and abiotic reduction would occur. Similarly, Bunge *et al.* observed that increasing the biomass:Pd(ii) ratio in Pd/*Cupriavidus necator* resulted in a higher fraction of periplasmic Pd(0) compared to extracellular Pd(0). This corresponded to a loss of catalytic activity in the production of hydrogen from hypophosphite.^97^ De Windt *et al.* observed that the highest CDW : Pd ratio (10 : 1) tested produced the smallest nanoparticles, mean size <20 nm, with the highest catalytic activity for perchlorate reduction.^30^ The perchlorate reduction rate fell sharply when catalysed by larger nanoparticles synthesised at lower CDW : Pd ratios. Some authors have suggested that bio-Pd nanoparticles are susceptible to catalyst poisoning by sulfur containing amino acids, particularly in smaller particles prepared at higher CDW : Pd.^81,97^ On the other hand, due to the relatively low proportion of sulfhydryl binding sites in live cells, Pd–S formation in bio-Pd may actually be relatively low and this could even contribute to enhanced catalytic activity or selectivity.^98–101^

### 4.2 Bio-Pd in dissimilatory metal-reducing bacteria (DMRB)

**Fig. 1:**
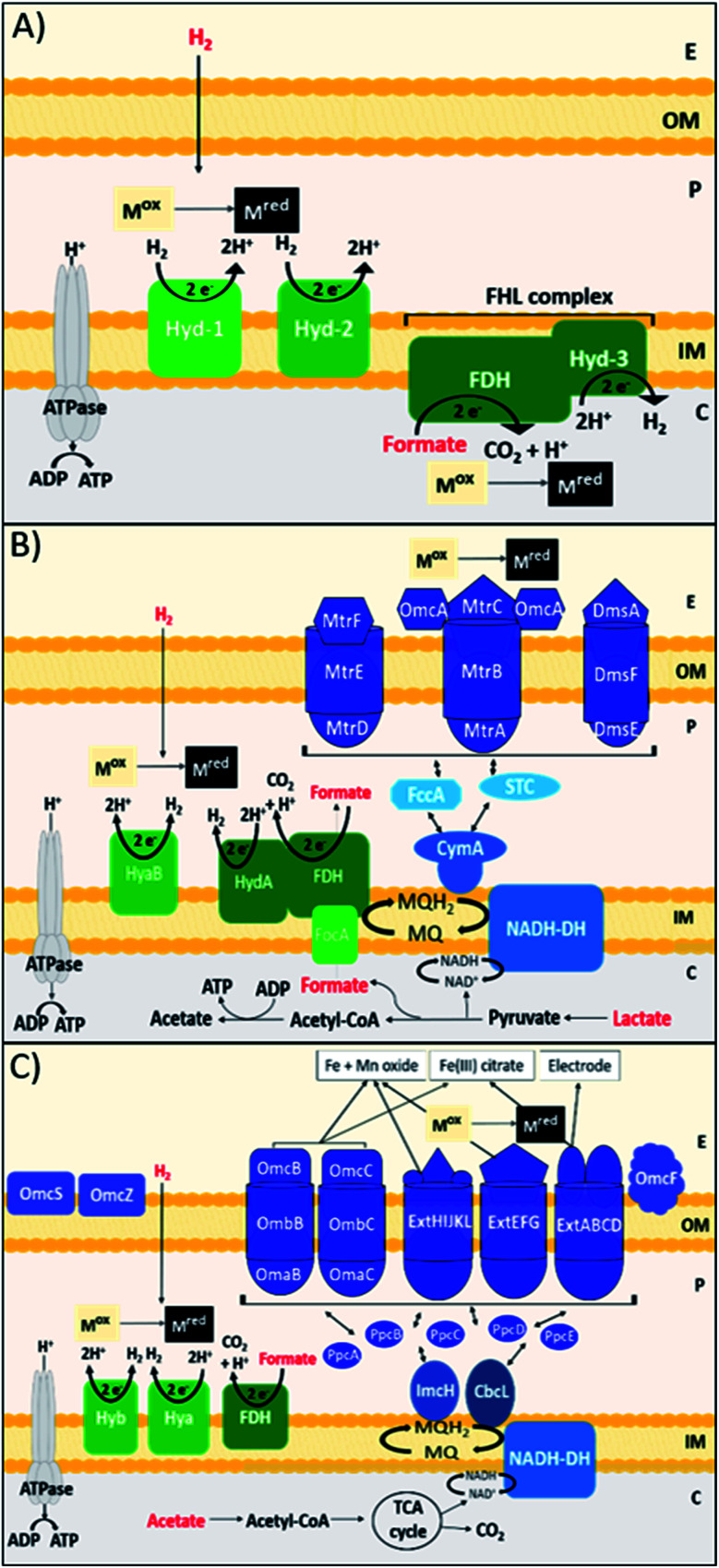
Schematic of metal bioreduction pathways suggested to reduce Pd(ii) in the outer and inner membranes of three Gram-negative bacteria (A) *Escherichia coli*, (B) *Shewanella oneidensis*, and (C) *Geobacter sulfurreducens*. Electron donors are in red, M^ox^ = oxidised metal species, M^red^ = reduced metal species, C = cytoplasm, IM = inner membrane, P = periplasm, OM = outer membrane, E = cell exterior, NADH-DH = NADH dehydrogenase, MQ = menaquinone pool, FHL = formate-hydrogen lyase complex, Omc = outer membrane cytochrome, Ppc = periplasmic cytochrome, [NiFe]-hydrogenase = Hyd-1, HyaB, Hyb. Nanowires are not shown as they are not implicated in Pd(ii) reduction.

DMRB obtain energy for growth in anaerobic sedimentary environments, by coupling the oxidation of organic electron donors to the reduction of metal species.^102^ DMRB naturally utilise minerals, such as insoluble Fe(iii) and Mn(iv) oxides, as terminal electron acceptors, and are directly involved in the biogeochemical cycling of carbon and other elements, including many metals.^103^ DMRB have been used to remediate metal and radionuclide contaminants, such as Cr(vi), U(vi), Tc(vii), from soil and aqueous environments, and can even be used in electricity production *via* the reduction of an anode surface in a microbial fuel cell.^104–107^ DMRB have been shown to reduce precious metal ions, such as Au(iii), Ag(i), Pt(iv) and Pd(ii), forming zerovalent metal nanoparticles.^19,108–111^ DMRB synthesise electron conductive pathways that run from the inner membrane to the surface of the outer membrane and beyond; these pathways are comprised primarily of c-type cytochromes.^112^ In terms of Pd(ii) bioreduction, DMRB can oxidise a wide diversity of electron donors, and this can affect the reduction pathway, nanoparticle localisation, and the rate of reduction for a particular metal species, potentially affecting the properties of nanoparticles produced. *Shewanella* species are marine facultative anaerobic bacteria that thrive at redox interfaces.^113^*Shewanella* species perform the recovery and formation of nanoparticles of precious metals such as Au, Ag, Pt, Rh and In,^108,114–117^ and have produced catalytically active bio-Pd nanoparticles.^30,96,118–120^ A key model *Shewanella* species for study is *Shewanella oneidensis* MR-1, which utilises lactate, formate and hydrogen most effectively as electron donors for metal reduction, *via* reasonably well-understood metabolic pathways (Fig. 1B).^121–123^ H_2_ and formate consistently produce faster rates of metal reduction in *S. oneidensis* than lactate, through the periplasmic [NiFe]-hydrogenase hyaB, and FDH enzymes.^106,124,125^ When lactate is utilised as the electron donor, electrons are passed to the outer membrane through the Mtr pathway, in particular the MtrCAB conduit that spans the outer membrane.^132,133^*S. oneidensis* utilises these outer membrane cell surface c-type cytochromes, MtrC and OmcA, as well as extracellular nanowires, to transfer electrons to oxidised metal species.^107,134,135^ Another model genus of DMRB is *Geobacter*, exemplified by *G. sulfurreducens*, an obligatory anaerobic Gram-negative bacterium found in the subsurface.^136^*G. sulfurreducens* transfers electrons to extracellular metal species *via* a diverse array of c-type cytochrome protein pathways, as well as through conductive extracellular pili nanowires (Fig. 1C).^137,138^*G. sulfurreducens* commonly utilises H_2_, formate and acetate as electron donors for bioreduction.^139^

De Windt *et al.* found that *S. oneidensis* is capable of reducing Pd(ii) using H_2_, formate, lactate, pyruvate and ethanol as electron donors. H_2_ was the most effective electron donor resulting in complete removal of Pd(ii) from solution with no significant decrease in the viability of cells. Formate and lactate were also effective, removing over 90% of Pd(ii).^19^*G. sulfurreducens* has been shown to reduce Pd(ii) using H_2_, formate, and acetate.^111,129,140^ Whereas H_2_ and formate driven reduction likely occurs due to the periplasmic [NiFe]-hydrogenase HyB, and periplasmic FDH enzymes,^129,139,140^ the outer membrane terminal reductase of acetate-driven Pd(ii) bioreduction in *G. sulfurreducens* has not yet been identified. A transcriptional analysis found that many of the outer membrane cytochromes in *G. sulfurreducens* that are important in Fe(iii) reduction, such as OmcB, OmcC, OmcS and OmcZ were downregulated during Pd(ii) reduction.^141–143^

Studies have shown that metal nanoparticles can be reduced at both the inner and outer membranes of these bacteria. For example, in *G. sulfurreducens* the acetate driven bioreduction of Ag(i) produced Ag(0) nanoparticles at the outer membrane, whereas the H_2_ driven bioreduction of Au(iii) produced Au(0) nanoparticles at the inner membrane.^144^ Dundas *et al.* used a *S. oneidensis* ΔHydA/ΔHyaB double hydrogenase mutant, with lactate as the electron donor, to confine bio-Pd to the outer membrane, whereas a ΔMtrC/ΔOmcA double deletion mutant confined bio-Pd to the periplasm.^145^ Yang *et al.* found that exposing *S. oneidensis* to the NADH-dehydrogenase inhibitor rotenone during formate driven Pd(ii) bioreduction also confined bio-Pd to the periplasm.^146^ In general, periplasmic Pd(0) nanoparticles are smaller than outer membrane Pd(0) NPs, for example Dundas *et al.* measured periplasmic Pd(0) to be <25 nm, compared to extracellular particles that were mostly 30–60 nm.^145^ Therefore the apparent trade-off between nanoparticle localisation, size, and exposed surface area due to choice of electron donor requires further study.

Another factor to consider in DMRB is the use of electron shuttles. *S. oneidensis* can perform indirect electron transfer *via* the secretion of flavins, such as riboflavin (RF) and flavin mononucleotide (FMN), which enhance extracellular electron transfer.^147,148^ Similarly, Okamoto *et al.* have shown that *G. sulfurreducens* cells secrete and utilise flavins as a bound cofactor in outer membrane c-type cytochromes.^149^ Dundas *et al.* found that either RF or FMN caused the number of Pd nanoparticles at the outer membrane to increase and the Pd nanoparticle size to decrease, although the bioreduction rate was slightly slower than the parent strain.^145^ The electron shuttle anthraquinone 2,6-disulfonate (AQDS) has been utilised to enhance the rate of Pd(ii) bioreduction in both *S. oneidensis* and *G. sulfurreducens*, producing smaller nanoparticles with enhanced catalytic activity.^35,72,129^

## 5. Industrial applications of bio-Pd

Pd/C is the most commonly used heterogeneous catalyst in industry, and recently the applications of Pd/C catalysts have been expanded.^4^ Bio-Pd shares some common features with abiotically produced Pd/C; both can be synthesised *via* the reduction of a Pd(ii) salt in the presence of the support, both contain crystallites roughly in the size range 2 to >20 nm, and Pd may vary in oxidation state in both depending on the degree of reduction.^15^ With respect to catalyst testing, so far bio-Pd studies have focused on hydrogenation and C–C coupling reactions.

### 5.1 Hydrogenation reactions

Hydrogenation reactions typically involve the addition of a pair of hydrogen atoms to a molecule *via* the reduction of double or triple bonds, which requires the presence of a catalyst. The chemistry is longstanding; Sabatier won the Nobel Prize in Chemistry in 1912 for using “finely disintegrated metals for the hydrogenation of organic compounds”,^150^ and currently, catalytic hydrogenations are responsible for the production of around 10–20% of industrial chemicals.^151^ Palladium nanoparticles are effective catalysts used for hydrogenation, selective hydrogenation, and hydrogenolysis reactions (Table 2) due to their high activity and hydrogen adsorption capacity.^11^ Industrially, catalytic hydrogenations are used to produce margarine,^152^ vitamins,^153–155^ and pharmaceuticals.^156^ Amongst many other examples, Pd/C catalysts are used in the *N*-debenzylation step of the synthesis of the anti-depressant nebivolol, and in the carbenzyloxy removal step of the antiviral drug valaciclovir.^157^

**Table 2:** Overview of bio-Pd systems catalysing hydrogenation, selective hydrogenation, and hydrogenolysis reactions, including the: species of bacteria, electron donor used for bioreduction, enzymatic reduction pathway, reaction catalysed in the study, reaction time, conversion, and yield of desired product (n/a = not applicable). Values designated with ∼ indicates data not explicitly stated in study but assessed from figures

Bacteria	Electron donor	Proposed reduction pathway	Catalytic application	Reaction time (hours)	Conversion (%)	Yield (%)	Reference
*Arthrobacter oxidans*	H_2_	Hydrogenase + abiotic	Selective hydrogenation of 2-butyne-1,4-diol	4	∼75	∼99	160
*Bacillus sphaericus*	H_2_	Hydrogenase + abiotic	Hydrogenation of itaconic acid	1	92.3	n/a	158
*Desulfovibrio desulfuricans*	H_2_	FDH + hydrogenase + abiotic	Dechlorination of 2-chlorophenol	2	∼16	n/a	92
				2	∼30		
	Formate		Dechlorination of 2,3,4,5-tetrachlorobiphenyl	2	10.4		
*Desulfovibrio desulfuricans*	H_2_	Hydrogenase + abiotic	Hydrogenation of itaconic acid	1	93.3	n/a	158
			Hydrogenation of 4-azidoaniline	3	84	n/a	126
			Selective hydrogenation of 3-nitrostyrene	2	81	74	126
			Hydrogenolysis of 1-bromo-2-nitrobenzene	2.25	10	10	126
			Selective hydrogenation of 2-butyne-1,4-diol	4	∼90	∼80	159
			Selective hydrogenation of 2-pentyne	5	100	∼55	25
			Upgrading crude bio-oil	4	77	n/a	162
*Desulfovibrio vulgaris*	H_2_	FDH + hydrogenase + abiotic	Dechlorination of 2-chlorophenol	2	∼9	n/a	92
	Formate		Dechlorination of 2,3,4,5-tetrachlorobiphenyl	2	∼18		
				2	3		
*Desulfovibrio* sp. ‘O7’	H_2_	FDH + hydrogenase + abiotic	Dechlorination of 2-chlorophenol	2	∼16	n/a	92
	Formate			2	∼15		
*Escherichia coli* (bio-Pt)	H_2_	Hydrogenase + abiotic	Selective hydrogenation of 2-butyne-1,4-diol	5	100	∼10	163
			Selective hydrogenation of 2-methyl-3-butyn-2-ol	3	∼55	∼38	161
			Selective hydrogenation of 4-octyne	4.15	∼90	∼63	
			Selective hydrogenation of 2-pentyne	100	∼71	∼60	
*Escherichia coli*	H_2_	Hydrogenase + abiotic	Selective hydrogenation of 2-pentyne	0.83	100	55	164
			Selective hydrogenation of soybean oil	5	45.5	∼41	
					78.4		
*Rhodobacter capsulatus*	H_2_	Hydrogenase + abiotic	Selective hydrogenation of 2-butyne-1,4-diol	5	100	∼65	160
*Shewanella oneidensis*	Formate	FDH + hydrogenase + abiotic	Dechlorination of 2,3,4-trichloro biphenyl	5	100	32	30
			Dechlorination of lindane (γ-hexachlorocyclohexane)	24	100	100	165

#### 5.1.1 Bio-Pd hydrogenation

Bio-Pd was first assessed as a hydrogenation catalyst for the conversion of itaconic acid to 2-methylsuccinic acid (Table 2). Bio-Pd was synthesised in cells of Gram-negative *D. desulfuricans* and Gram-positive *B. sphaericus*.^158^ Different Pd(ii) loadings produced bio-Pd with different catalytic activities for the hydrogenation of itaconic acid, with 5% Pd/*D. desulfuricans* (wt/dry wt) and 2% Pd/*B. sphaericus* achieving 99% and 97% conversions, respectively, of that achieved by a 5% Pd/C (wt/wt) commercial catalyst after 60 minutes. In a following study, 5% Pd/*D. desulfuricans* catalysed the hydrogenation of 4-azidoaniline hydrochloride to 1,4-phenylenediamine with an 84% conversion. This was comparable to a 10% Pd/C commercial catalyst that converted 73% of the starting material.^126^ The authors noted that periplasmic bio-Pd held below the outer membrane likely caused mass transfer limitations to the catalytic surface.

#### 5.1.2 Bio-Pd selective hydrogenation

Bio-Pd nanoparticles have been shown to catalyse selective hydrogenations with greater selectivity towards the desired product than some conventional commercial catalysts. Bio-Pd was used to catalyse the selective hydrogenation of 3-nitrostyrene to 1-ethyl-3-nitrobenzene in methanol (Table 2). A 5% Pd/*D. desulfuricans* (wt/dry wt) catalyst converted 81% of the starting material, producing 74% of the desired product and 7% of the fully hydrogenated product, 1-ethyl-3-aminobenzene.^126^ In comparison a 10% Pd/C (wt/wt) commercial catalyst produced 73% of the fully hydrogenated product. Pd/*D. desulfuricans* had selective activity towards the C=C bond, whereas Pd/C reduced the –NO_2_ group. In a follow-up study, Pd/*D. desulfuricans* gave superior selectivity over a 5% Pd/Al_2_O_3_ (wt/wt) commercial catalyst for the selective hydrogenation of 2-butyne-1,4-diol, performed in 2-propanol.^159^ A 20% Pd/*D. desulfuricans* (wt/dry wt) catalyst converted over 80% of the alkyne to the desired alkene product, 2-butene-1,4-diol. In comparison, the commercial catalyst produced over 80% of the undesired alkane product, 2-butane-1,4-diol. Interestingly, 5% Pd/*D. desulfuricans* converted less of the starting material and produced less alkene (∼60%) than 20% Pd/*D. desulfuricans*, both at a catalyst loading of 0.205 mol% Pd.^159^ The same reaction was catalysed by bio-Pd from the Gram-positive soil bacterium *Arthrobacter oxidans* and the Gram-negative photosynthetic bacterium *Rhodobacter capsulatus*.^160^ The bio-Pd samples achieved maximum selectivities of 0.98 and 1.0 for 5% Pd/*A. oxidans* and 5% Pd/*R. capsulatus*, respectively. A 5% Pd/Al_2_O_3_ commercial catalyst gave a fast rate of reaction but was not selective, achieving 0.67 selectivity at 73% conversion, compared to ∼0.85 for 5% Pd/*A. oxidans* at the same conversion.^160^ The hydrogenation rate in bio-Pd may have been slower due to the mass transfer limitations of reactants diffusing through the outer membrane, which may have helped to prevent overhydrogenation. In addition, 25% Pd/*A. oxidans* gave a slower reaction rate but higher selectivity at 100% conversion than 5% Pd/*A. oxidans*, which was likely due to size effects. Larger bio-Pd particles at the higher metal loading would have lower activity, possessing fewer low coordination number sites such as terraces and edges, preventing overhydrogenation. Bio-Pt nanoparticles on *E. coli* were shown to possess comparable selectivity to a reduced Pt/C catalyst for the selective hydrogenation of various alkynes.^161^ In the selective hydrogenation of 2-butyne-1,4-diol using a 20% Pt/*E. coli* (wt/dry wt) catalyst, the butenediol selectivity was increased significantly when the bio-Pt was chemically processed to remove most of the biomass, although the reaction rate fell considerably.^163^ These results suggest that removing biomass from the nanoparticles may liberate defect sites on the metal surface that favour formation of the alkene product. Bio-Pd was also investigated for the selective hydrogenation of 2-pentyne, where it displayed superior selectivity over a commercial Pd/Al_2_O_3_ catalyst. Pd/*D. desulfuricans* retained a higher selectivity for 2-pentene than Pd/Al_2_O_3_ at alkyne conversions above 70%, although 5% Pd/*D. desulfuricans* (wt/dry wt) only produced 30% of the reaction rate of 5% Pd/Al_2_O_3_ (wt/wt).^25^ At 92.5% alkyne conversion, 5% Pd/*D. desulfuricans* gave a pentene/pentane ratio of 3.3 and a *cis*/*trans* ratio of 2.5. In comparison, Pd/Al_2_O_3_ achieved lower ratios of 2.0 and 2.0, respectively. At a higher Pd(ii) loading, 25% Pd/*D. desulfuricans* achieved a faster rate of reaction and higher selectivity than 5% Pd/*D. desulfuricans.* Alumina may adsorb excess hydrogen that acts as a local hydrogen reservoir, causing the overhydrogenation of 2-pentene to pentane.^166^ Cell biomass, on the other hand, may be more inert to hydrogen adsorption and would not experience this affect.

In a following study, 2% Pd/*E. coli* (wt/dry wt) achieved a *cis*/*trans* 2-pentene ratio of 2.8 at 100% 2-pentyne conversion, compared to 0.7 for a 2% Pd/Al_2_O_3_ commercial catalyst.^164^ A 5% Pd/*E. coli* catalyst gave a threefold higher rate of reaction than 2% Pd/*E. coli,* but with a lower *cis*/*trans* ratio of 1.6. The hydrogenation rate constant of the C≡C was calculated to be 7.6 times higher than of the C=C bond, and 2-pentyne adsorption was measured to be much stronger than 2-pentene adsorption to the Pd surface.

Zhu *et al.* also investigated the solvent-free selective hydrogenation of soybean oil by bio-Pd.^164^ A 5 wt% Pd/*E. coli* catalyst required over 5 hours to convert around 50% of the reactant oil substrate, whereas 5 wt% Pd/Al_2_O_3_ converted over 60% in 1 hour. The Pd/Al_2_O_3_ catalyst reached a peak concentration of 1.07 mol L^−1^ of the desired alkene product, *cis*-C18 : 1, but this fell to less than half by the end of the reaction, with the major product being the fully hydrogenated alkane. On the other hand, 5% Pd/*E. coli* recorded a peak concentration of 1.03 mol L^−1^ of *cis*-C18 : 1, which did not fall throughout the reaction, and only low concentrations of undesired products were produced. When 5% Pd/*D. desulfuricans* was tested for the same reaction it produced very similar hydrogenation products to Pd/*E. coli*.

#### 5.1.3 Bio-Pd hydrogenolysis

The hydrogenolysis of pollutant organohalogen compounds can be performed on palladium catalysts using hydrogen or organic acids such as formate.^167^ Bio-Pd has been shown to catalyse the hydrodehalogenation of compounds such as polychlorinated biphenyls (PCBs) and trichloroethylene (Table 2).^168–170^ An early study by Baxter-Plant *et al.* demonstrated the dechlorination of 2-chlorophenol and various PCBs using bio-Pd from three different *Desulfovibrio* species.^92^ Pd/*D. desulfuricans* repeatedly produced the most active bio-Pd for all the tested reactions, including successfully dechlorinating a hexachlorinated PCB. Interestingly, bio-Pd produced using formate as the electron donor was repeatedly more active than bio-Pd from H_2_-driven bioreduction across all three species of bacteria. Pd/*S. oneidensis* catalysed the complete dehalogenation of all 3 isomers of lindane (hexachlorocyclohexane, HCH) to benzene within 24 hours.^165^ In comparison, a commercial powdered Pd(0) catalyst could not completely remove the substrate after 48 hours. The reductive debromination of 1-bromo-2-nitrobenzene to nitrobenzene was catalysed by 5% Pd/*D. desulfuricans*, which only converted 10% of the substrate, but it was highly selective only yielding the desired product, nitrobenzene.^126^ A 10% Pd/C catalyst converted 92% of the starting material, but only catalysed the complete reduction of 1-bromo-2-nitrobenzene to the undesired product, aniline.

### 5.2 C–C coupling reactions

**Scheme 1:**
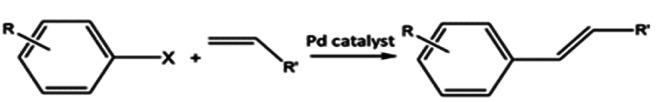
General reaction scheme of the Heck reaction. Where X = I, Br, Cl.

**Scheme 2:**
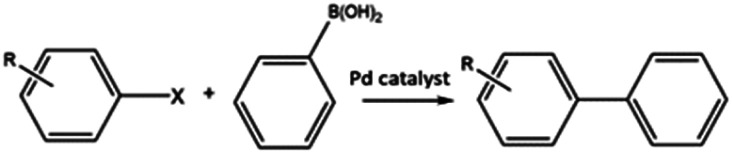
General reaction scheme of the Suzuki reaction. Where X = I, Br, Cl.

C–C bond forming coupling reactions have become an indispensable tool in organic synthesis and have led to a step change in the synthesis of complex drug molecules, enabling the parallel synthesis of key precursor components that can be coupled together at a later stage.^171^ Heck and Mizoroki discovered the coupling of aryl or vinyl halides with activated alkenes in the presence of a base, forming a C–C bond to produce a substituted alkene (Scheme 1).^172,173^ The Heck reaction has been used in industrial applications to synthesise products such as the anti-inflammatory drug naproxen,^174^ the asthma drug Singulair^175^ and the herbicide prosulfuron.^176^ The Suzuki–Miyaura C–C coupling reaction was later developed in 1979, which couples a boronic acid or ester with organic halides to produce a biaryl derivative (Scheme 2).^177^ The Suzuki reaction has also been used extensively on an industrial scale, such as to manufacture the fungicide Boscalid,^178^ and for a key step in the natural product (+)-discodermolide, a potent cancer cell growth inhibitor.^179^

Although Heck used homogeneous Pd(ii) acetate as his original catalyst, it was noted that heterogeneous Pd/C catalysts were active for the reaction, albeit with lower rate and yields; later studies confirmed this and showed some activity for aryl chlorides.^180^ Pd/C catalysts with high activity for Heck reactions of nonactivated bromobenzene were reported, with optimized conditions allowing Pd concentrations as low as 0.005 mol%.^54^ The authors found that high Pd dispersion, low degree of Pd reduction, high water content, and uniform Pd impregnation combined to give the most active Pd/C catalyst. Pd/C was first shown to catalyse Suzuki coupling reactions by Marck *et al.*^181^ and have been reported for the construction of biaryl derivatives in good to excellent yields, and with good reusability.^182,183^ The C–C coupling of aryl bromides was demonstrated in Heck, Suzuki, and Sonogashira couplings.^184^

#### 5.2.1 Bio-Pd Heck coupling

Bio-Pd was first explored as a C–C coupling catalyst for the Heck coupling of iodobenzene with ethyl acrylate (Table 3), and the reaction was carried out in dimethylformamide (DMF), with trimethylamine as base, at a catalyst loading of 0.205 mol%, at 120 °C for 4 hours under N_2_ atmosphere.^159^ 5% Pd/*D. desulfuricans* (wt/dry wt) performed comparably to a commercial 5% Pd/C catalyst with both achieving 98% conversion to ethyl cinnamate after 2 hours. A higher metal loading of 20% Pd/*D. desulfuricans* achieved around 90% conversion. In a following study, the catalyst loading was increased to 0.5 mol%, resulting in a product conversion of 96.4% after 2 hours, and an initial rate of 57 mmol min^−1^ g^−1^ Pd.^26^ In comparison, a 1 mol% loading of tetraalkylammonium-stabilised colloidal Pd nanoparticles achieved 99.5% conversion after 4 hours, with a lower initial rate of 24 mmol min^−1^ g^−1^ Pd. The Heck coupling of iodobenzene with styrene was also performed under the same conditions, with 5% Pd/*D. desulfuricans* again achieving the highest product conversion of 85%, and an initial rate of 30 mmol min^−1^ g^−1^ Pd. The colloidal Pd nanoparticles only achieved 71% conversion after 5 hours, with an initial rate of 14 mmol min^−1^ g^−1^ Pd.^26^ In both Heck couplings, increasing the catalyst load resulted in lower final conversions. A higher metal loading of 25% Pd/*D. desulfuricans* achieved lower final conversions than 5% Pd/*D. desulfuricans*, indicating the size of bio-Pd nanoparticles may have influenced the reaction.

**Table 3:** Overview of Heck couplings by bio-Pd including the: species of microbe, electron donor used for bioreduction, enzymatic reduction pathway, aryl halide, coupling partner, reaction time, and conversion of the reaction. Values designated with ∼ indicates data not explicitly stated in study but assessed from figures

Microbe	Electron donor	Proposed reduction pathway	Aryl halide	Coupling partner	Reaction time (hours)	Conversion (%)	Reference
*Arthrobacter oxidans*	H_2_	Hydrogenase + abiotic	Iodobenzene	Ethyl acrylate	2	51	83
				Styrene	4	∼65	
*Cupriavidus metallidurans*	H_2_	Hydrogenase + abiotic	Iodobenzene	Ethyl acrylate	2	∼70	83
				Styrene	4	∼70	
*Cupriavidus necator*	H_2_ or formate	FDH + hydrogenase + abiotic	Iodobenzene	*n*-Butyl acrylate	12	97	24
			4-Iodobenzonitrile		24	100	
			4-Chloro-iodobenzene		24	100	
			4-Iodobenzaldehyde		24	88	
			4-Iodotoluene		24	98	
			Methyl 4-iodobenzoate		24	86	
			2-Iodobenzaldehyde		24	86	
			5-Iodo-2-methoxybenzoate		24	81	
			1-Iodo-4-methoxybenzene		24	96	
			4-Bromobenzonitrile		24	97	
			1-Bromo-4-nitrobenzene		24	88	
			1-Bromo-4-(trifluoromethyl)benzene		24	53	
*Desulfovibrio desulfuricans*	H_2_	Hydrogenase + abiotic	Iodobenzene	Ethyl acrylate	2	98	26, 83 and 159
					5	96.4	
				Styrene	2	∼80	
					4	80	
*Escherichia coli*	H_2_	Hydrogenase + abiotic	Iodobenzene	Ethyl acrylate	2	78	83
				Styrene	4	75	
			4-Bromoacetophenone	Ethyl acrylate	5	54	
			3-Chlorotoluene	Ethyl acrylate	5	0	
*Micrococcus luteus*	H_2_	Hydrogenase + abiotic	Iodobenzene	Ethyl acrylate	2	∼65	83
				Styrene	4	∼65	
*Pseudomonas putida*	Formate	FDH + hydrogenase + abiotic	Iodobenzene	*n*-Butyl acrylate	24	99	24
			1-Iodo-4-methoxybenzene		24	96	
			4-Bromobenzonitrile		24	88	
*Phanerochaete chrysosporium*	None	Putative fungal enzymes	Iodobenzene	Styrene	3	95	68
*Serratia* sp.	H_2_	Hydrogenase + abiotic	Iodobenzene	Ethyl acrylate	2	∼85	83
				Styrene	4	∼65	
*Shewanella oneidensis*	H_2_	Hydrogenase + abiotic	Iodobenzene	Ethyl acrylate	2	>90	83
				Styrene	4	60	

Søbjerg *et al.* tested bio-Pd as a catalyst for the Heck couplings of various aryl halides with *n*-butylacrylate (Table 3) in the presence of DMF and Na_2_CO_3_, at a catalyst loading of <1 mol%, at 80 °C for 12–24 hours.^24^ Bio-Pd was synthesised on cells of two Gram-negative Proteobacteria, *Cupriavidus necator*, which formed mostly small, well dispersed particles <10 nm, and *Pseudomonas putida*, which formed large Pd aggregates >100 nm. Both bio-Pd catalysts successfully converted activated and nonactivated aryl iodides in high yields, performing comparably to a commercial Pd nanopowder (<25 nm). Neither bio-Pd catalyst catalysed the coupling of non-activated aryl bromides or activated aryl chlorides. The significant size differences between Pd/*C. necator* and Pd/*P. putida* affected their activities for the Heck reaction, achieving conversions of 97% and 88%, respectively, for the coupling of the activated aryl bromide 4-bromobenzonitrile with *n*-butylacrylate. It is worth noting the reaction was only catalysed with the addition of tetrabutylammonium bromide (TBAB), which can stabilise Pd nanoparticles in solution and acts as a phase transfer catalyst.^24^ The requirement of TBAB indicates active Pd species may leach into solution from bio-Pd, a common mechanism for supported Pd catalysts in Heck and Suzuki reactions.^12^

Deplanche *et al.* showed that the species of bacteria had a significant effect on the ability of bio-Pd to catalyse the Heck coupling of phenyl iodide with styrene, and with ethylacrylate.^83^ Gram-negative species generally outperformed Gram-positive species, with Pd/*E. coli*, Pd/*D. desulfuricans*, and Pd/*C. metallidurans* achieving the highest conversions.

All three catalysts gave strongly biphasic reaction profiles, for example Pd/*E. coli* catalysed 75% of the coupling of phenyl iodide and ethylacrylate in the first 15 minutes, followed by a plateau in conversion. Bio-Pd could also show significant disparities between reactions. Pd/*S. oneidensis* displayed a 30 minute lag phase before converting 90% of the coupling of phenyl iodide and ethylacrylate, but gave one of the lowest conversions of 60% for the coupling of phenyl iodide and styrene. 2.5% Pd/*E. coli* (wt/dry wt) gave a 78% conversion for the coupling of phenyl iodide and ethyl acrylate, achieved a 54% conversion of the aryl bromide, 4-bromoacetophenone, but was not able to catalyse the coupling of the aryl chloride, 3-chlorotoluene.^83^ Recently, the fungus *Phanerochaete chrysosporium* synthesised bio-Pd of 10–14 nm, which was assessed for the Heck coupling of iodobenzene and styrene.^68^ Pd/*P. chrysosporium* produced 95% conversion to stilbene after 120 minutes, which was comparable to a 5% Pd/C commercial catalyst.

#### 5.2.2 Bio-Pd Suzuki coupling

Søbjerg *et al.* first assessed bio-Pd for the Suzuki coupling of various aryl halides with phenylboronic acid in the presence of Na_2_CO_3_, TBAB and EtOH/H_2_O, with a catalyst loading of <2 mol%, for 6–24 hours at 50–80 °C.^24^ Pd/*C. necator* successfully catalysed the conversion of several activated and nonactivated aryl iodides, at final product yields ranging from 79–100% (Table 4). Pd/*P. putida* gave 100% product yield for the coupling of *p*-iodoanisole and phenylboronic acid. Attempts to catalyse the coupling of the aryl bromide, 4-bromobenzonitrile using bio-Pd were unsuccessful, whereas a commercial Pd nanopowder achieved a 54% conversion. In a later study the Suzuki coupling of iodoanisole and phenylboronic acid was performed under the same conditions, using Pd/*C. necator* and Pd/*S. sciuri*.^31^ Bio-Pd was synthesised at different CDW : Pd ratios, and at the lowest ratio almost all of the bio-Pd(0) nanoparticles were measured to be >100 nm. These larger agglomerated particles had the highest catalytic activity, achieving complete substrate conversions. Conversely, at the highest CDW : Pd ratio all the particles were measured to be <25 nm, and no catalytic activity was observed.

**Table 4:** Overview of Suzuki couplings by bio-Pd including the: species of microbe, electron donor used for bioreduction, enzymatic reduction pathway, aryl halide coupled to phenyl boronic acid, reaction time, and conversion of the reaction

Bacteria	Electron donor	Proposed reduction pathway	Aryl halide	Reaction time (hours)	Conversion (%)	Reference
*Cupriavidus necator*	Formate	FDH + hydrogenase + abiotic	Iodobenzene	6	86	24
			4-Iodoanisole	6	100	
			4-Iodotoluene	6	96	
			4-Chloro-iodobenzene	6	60	
			4-Iodobenzonitrile	6	84	
			4-Iodoacetophenone	6	97	
			4-Iodobenzaldehyde	6	79	
			2-Iodobenzaldehyde	6	75	
			3-Iodo-*N*-methylbenzamide	16	97	
			4-Methoxy-iodobenzene	6	100	
			Methyl 4′-iodo-4-methoxybiphenyl-3-carboxylate	6	89	
			4-Bromobenzonitrile	16	0	
*Cupriavidus necator*	H_2_	Hydrogenase + abiotic	4-Iodoanisole	20	100	31
*Escherichia coli*	H_2_	Hydrogenase + abiotic	4-Bromoanisole	18	62	83
			1-Bromo-4-(trifluoromethyl)benzene	18	90	
			4-Bromoacetophenone	18	23	
			2-Bromopyridine	18	0	
			4-Chloroanisole	24	17	
			3-Chlorotoluene	18	0	
*Pseudomonas putida*	Formate	FDH + hydrogenase + abiotic	4-Iodoanisole	6	100	24
			4-Bromobenzonitrile	6	0	
*Staphylococcus sciuri*	H_2_	Hydrogenase + abiotic	4-Iodoanisole	20	100	31

Deplanche *et al.* assessed 2.5% Pd/*E. coli* (wt/dry wt) for the Suzuki coupling of different aryl halides with phenylboronic acid in the presence of Na_2_CO_3_, water, ethanol, and John-Phos ligand, at a catalyst loading of 0.1 mol%, for 18 hours at 80 °C.^83^ The Suzuki couplings were unreactive without a monophosphine (John-Phos) ligand present, which can stabilise Pd species leached into solution, which may suggest a homogeneous reaction mechanism. Pd/*E. coli* displayed good activity for the activated substrate 1-bromo-4-(trifluoromethyl)benzene, and for 4-bromoanisole, whereas the activated substrate 4-bromoacetophenone produced only a moderate conversion. No reaction was observed for 2-bromopyridine or for the aryl chloride 3-chlorotoluene, and a modest 17% yield was achieved for 4-chloroanisole.

## 6. Microbial bimetallic nanoparticles

Bimetallic nanoparticles have been shown to possess superior catalytic activity and selectivity over monometallic nanoparticles in industrially relevant reactions such as C–C coupling reactions and hydrogenations.^185–187^ Introducing a second metal phase to the nanoparticle structure can change its properties, such as the surface composition, the geometry of surface adsorption sites, and the electronic properties of the catalyst.^188^ Furthermore, altering the ratio of the two metals enables fine-tuning of these properties.

Microbial bimetallic nanoparticles are a relatively new addition to the field with significant potential, and have demonstrated far superior catalytic properties to monometallic bio-Pd (Table 5). Fine tuning of the nanostructure of these particles, such as developing well-defined core–shell structures, along with better control of particle size, and identification of the catalytically active species provides the possibility of achieving even better catalytic performance.

**Table 5:** Overview of bimetallic bio-Pd-based systems catalysing various reactions, including the: species of bacteria, dual metals reduced, electron donor used for bioreduction, enzymatic reduction pathway, and the reaction catalysed in the study

Bacteria	Metals	Electron donor	Proposed reduction pathway	Catalytic application	Reference
*Bacillus benzeovorans*	Pd + Pt	H_2_	Hydrogenase + abiotic	Upgrading heavy oil	195
	Pd + Ru			Transfer hydrogenation of 5-HMF	196
*Cupriavidus necator*	Pd + Au	Formate	FDH + hydrogenase + abiotic	4-Nitrophenol reduction	197
*Desulfovibrio desulfuricans*	Pd + Au	H_2_	Hydrogenase + abiotic	Benzyl alcohol oxidation	34 and 191
	Pd + Pt			Upgrading heavy oil	195
*Escherichia coli*	Pd + Au	H_2_	Hydrogenase + abiotic	Benzyl alcohol oxidation	34 and 192
	Pd + Pt			Cr(vi) reduction	20
				Selective hydrogenation of 2-pentyne	20
				Selective hydrogenation of soybean oil	21
	Pd + Ru			Transfer hydrogenation of 5-HMF	198
*Shewanella oneidensis*	Pd + Au	H_2_	Hydrogenase + abiotic	Dechlorination of trichloroethene and diclofenac	33 and 199
		Formate	FDH + hydrogenase + abiotic	Suzuki coupling	32
		Lactate	OMC (MtrCAB)	Suzuki coupling	36
			OMC (MtrCAB) (biomagnetite)	Reduction of nitroaromatics	200
	Pd + Ag	Lactate	OMC (MtrCAB) (reduced GO)	4-Nitrophenol reduction	201
			OMC (MtrCAB)	Suzuki coupling	36
	Pd + Pt	Formate	FDH + hydrogenase + abiotic	4-Nitrophenol reduction	35 and 202

### 6.1 Bio-PdAu

Bio-PdAu was the first microbial bimetallic system reported. De Corte *et al.* found that bio-PdAu possessed different catalytic properties when synthesised *via* simultaneous or sequential reduction by *S. oneidensis*.^33^ Bio-PdAu produced from the simultaneous reduction of Pd(ii) and Au(iii) (PdAu/*S. oneidensis*) degraded 77.8% of the environmental contaminant diclofenac after 24 hours. Bio-PdAu produced from the sequential reduction of Au(iii) followed by Pd(ii) (AuPd/*S. oneidensis*), only degraded 36.5% of diclofenac. However, bio-PdAu produced from the sequential reduction of Pd(ii) followed by Au(iii) (PdAu/*S. oneidensis*) produced little catalytic activity. Simultaneously reduced PdAu/*S. oneidensis* also dechlorinated trichloroethylene (TCE) in 40 minutes, vastly outperforming sequentially reduced Au–Pd/*S. oneidensis*. Thin section TEM showed that simultaneously reduced PdAu/*S. oneidensis* possessed on average larger nanoparticles than sequentially reduced AuPd/*S. oneidensis*, with a mean particle size of 11.0 nm and 6.9 nm, respectively. μXRD showed a significant contraction of the simultaneously reduced PdAu/*S. oneidensis* lattice relative to Pd/*S. oneidensis*, suggesting the formation of unique crystalline domains of alloyed PdAu. This alloyed phase was only weakly present in sequentially reduced AuPd/*S. oneidensis*, which predominantly contained a mixture of monometallic Pd and Au. Therefore it appears the enhanced catalytic performance of PdAu/*S. oneidensis* resulted from structural rather than size differences. Chemically synthesised core–shell Au–Pd nanoparticles were shown to catalyse TCE degradation with rate constants up to 1000 times higher than for the alloyed structure of PdAu/*S. oneidensis*.^189^ The core–shell structure may allow Au atoms to withdraw electron density from Pd more effectively than an alloyed structure, thereby increasing the interaction potential of Pd with reactants.^190^

**Fig. 2:**
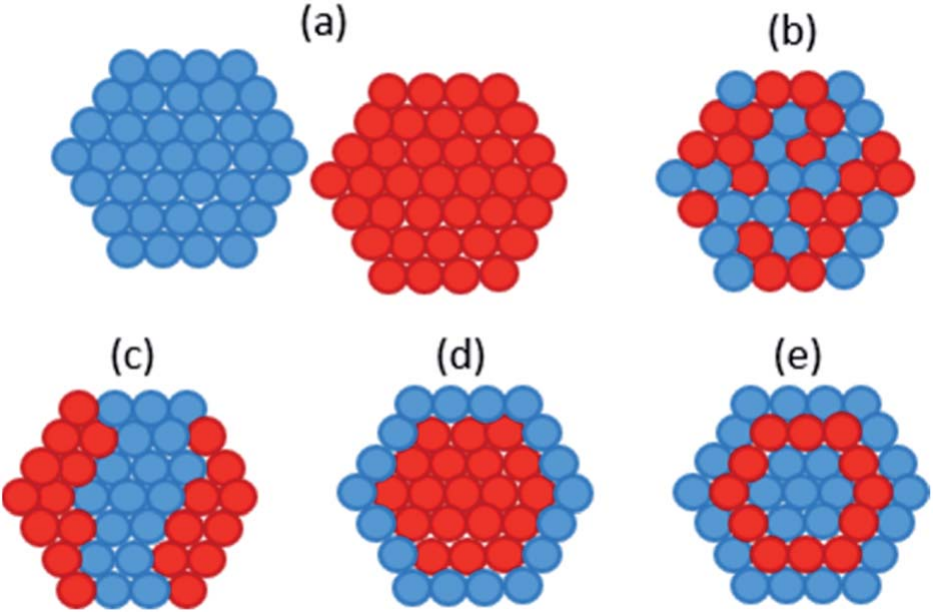
Different possible atomic structures of bimetallic nanoparticles (a) separate monometallic nanoparticles; (b) mixed alloys; (c) cluster-in-cluster; (d) core–shell; (e) multishell.

Deplanche *et al.* synthesised bio-PdAu using cells of D. desulfuricans and *E. coli via* the sequential reduction of Pd(ii) followed by Au(iii).^34^ When tested for the oxidation of benzyl alcohol, PdAu/*E. coli* gave a 35% conversion and 96% selectivity to the desired product benzaldehyde, and PdAu/*D. desulfuricans* gave 38% conversion and 95% selectivity. In comparison, a 5% Pd/C commercial catalyst only gave 25% conversion and 70% selectivity. In following studies characterising bio-PdAu,^191,192^ the authors found evidence of core–shell structure within bio-PdAu (Fig. 2d). They suggested the structure was formed from a sacrificial hydrogen mechanism, whereby Pd(0) surface atoms are oxidized to Pd(ii) ions by undergoing galvanic exchange with Au(iii) ions, which are then reduced to Au(0). The Pd(ii) ions would then be re-reduced to form an outer Pd(0) shell. However, this mechanism has been shown to form a ‘cluster-in-cluster’ structure (Fig. 2c) as opposed to core–shell.^193^ PdAu/*G. sulfurreducens* was synthesised by simultaneous reduction of Pd(ii) and Au(iii), which contained some larger particles with core–shell Au–Pd structure.^194^ The core–shell structure was attributed to the higher reduction potential of Au(iii), as slow reduction kinetics may have resulted in Au seeds initially forming onto which Pd shells can subsequently grow.

Kimber *et al.* synthesised PdAu/*S. oneidensis via* simultaneous reduction of Pd(ii) and Au(iii) and tested the resulting nanoparticles for Suzuki coupling reactions.^36^ EXAFS revealed that the bio-PdAu nanoparticles displayed increased bond lengths suggesting limited alloying of the metals, and there was also some indication of non-uniform core–shell structure in a minority of particles. PdAu/*S. oneidensis* outperformed monometallic Pd/*S. oneidensis* for the Suzuki coupling of phenylboronic acid and 4-bromoacetophenone, converting >99% after 24 hours. These results are in agreement with previous results, where PdAu/*S. oneidensis* was shown to be more active for the Suzuki coupling of various aryl iodides, and an aryl bromide, with arylboronic acids than Pd/*S. oneidensis*.^32^ The effect of different electron donors in *S. oneidensis* was apparent as lactate driven particles displayed a narrower size distribution and smaller average particle size than H_2_ driven PdAu/*S. oneidensis*, likely due to abiotic reduction by H_2_.^33,36^ In addition to structural effects the composition of bio-PdAu nanoparticles has been shown to affect its catalytic properties. De Corte *et al.* synthesised PdAu/*S. oneidensis* with varying Pd(ii) and Au(iii) concentrations, where a low Pd : Au ratio (50 : 1) produced the fastest removal rate of diclofenac.^33^ Deplanche *et al.* reported that a 2.5/2.5 wt% Au/Pd metal loading on cells of *E. coli* and *D. desulfuricans* gave the best conversion and selectivity for the oxidation of benzyl alcohol, over a 1/4 wt% Au/Pd loading.^34^

### 6.2 Bio-PdAg

Han *et al.* used *S. oneidensis* to simultaneously reduce graphene oxide (GO), Pd(ii), and Ag(i) from solution to form PdAg/rGO/*S. oneidensis*, which significantly outperformed Pd/*S. oneidensis* for the reduction of 4-nitrophenol.^201^ The optimal catalytic rate constant for 4-nitrophenol reduction of 0.241 min^−1^ was obtained at a Pd/Ag ratio of 1 : 1 and a 2 mg catalyst loading, which produced a 99% yield of 4-aminophenol. The authors postulated that the transfer of electrons from Ag to Pd occurred through the conductive rGO sheets, to form electron-rich regions in the rGO sheets between the two metals, which offered additional active sites for adsorption and reaction. Kimber *et al.* produced PdAg/*S. oneidensis via* simultaneous reduction of Pd(ii) and Ag(i), which significantly outperformed bio-PdAu and bio-Pd for the Suzuki coupling of phenylboronic acid and 4-bromoacetophenone.^36^ Bio-PdAg converted >99% after 5 hours, compared to 70% and 11% for bio-PdAu and Pd, respectively. Monometallic bio-Ag and bio-Au nanoparticles were not active for the reaction. PdAg/*S. oneidensis* coupled a broad range of arylboronic acids and aryl bromides in good to excellent yields, even achieving a double coupling of 3,5-dibromobenzaldehyde with phenylboronic acid. PdAg/*S. oneidensis* nanoparticles had a larger average particle size (11.2 nm) and size distribution than PdAu/*S. oneidensis*. Less bimetallic alloying was observed in the bio-PdAg than bio-PdAu, and EXAFS suggested that multiple Pd species were present in PdAg/*S. oneidensis*, including Pd–O and Pd–S shells.

### 6.3 Bio-PdPt

Tuo *et al.* synthesised PdPt/*S. oneidensis via* the simultaneous reduction of Pd(ii) and Pt(iv), at pH = 7 in mineral salts medium.^35^ Two populations of PdPt/*S. oneidensis* nanoparticles were produced; small spheres with an average size of 4.41 nm, or larger flower-shaped 60 nm particles. PdPt/*S. oneidensis* catalysed the reduction of 4-nitrophenol with a rate constant of 0.0316 min^−1^, and after 6 cycles retained over 92% conversion of 4-nitrophenol, despite the rate falling to 0.0078 min^−1^. Xu *et al.* produced PdPt/*S. oneidensis* using a similar protocol, however the bioreduction was performed in solutions of the metals dissolved in HCl at pH = 2.^202^ These PdPt/*S. oneidensis* nanoparticles consisted of predominantly small spheres with an average size of 13.2 nm, and no flower-shaped particles were produced. A much improved rate constant of 0.743 min^−1^ was observed for 4-nitrophenol reduction. XPS detected Pt(0) along with mainly Pd(0) and some Pd(ii). The enhanced catalytic properties observed were likely due to changes in the lattice structure, and enhanced electron transport between the two metals. Omajali *et al.* demonstrated that bio-Pd and bio-PdPt nanoparticles gave comparable results to a commercial NiMo/Al_2_O_3_ catalyst for the catalytic upgrading of heavy oil.^195^ Gram-negative *D. desulfuricans* and the Gram-positive bacterium *Bacillus benzeovorans* first reduced Pd(ii) to form bio-Pd, followed by the reduction of Pt(iv). The commercial catalyst produced a higher American Petroleum Institute (API) gravity than the bio-PdPt catalysts but was also hindered by more coking. In addition, bio-PdPt produced higher volumes of liquid fractions on average.

### 6.4 Bio-PdRu

Macaskie *et al.* also produced bio-PdRu bimetallic nanoparticles for the catalytic transfer hydrogenation of 5-hydroxymethyl furfural (HMF) to produce the “drop in” fuel 2,5-dimethylfuran (DMF).^196,203^ PdRu/*B. benzeovorans* and PdRu/*E. coli* were both synthesised by sequential reduction, first pre-seeding cells with bio-Pd, followed by reduction of Ru(iii). Multiple species of both metals were identified including: Pd(0), Pd(iv), Ru(iii), Ru(iv), Ru(vi), but not Ru(0). 2.5/2.5 wt% PdRu/*B. benzeovorans* (wt/dry wt) gave a 94.7% conversion and 55.5% yield of HMF and DMF, respectively.^196^ In comparison, 5/5 wt% PdRu/*E. coli* (wt/dry wt) gave a 100% conversion and 54.4% yield of HMF and DMF, respectively, which was comparable to a commercial 5% Ru/C (wt/wt) catalyst.^203^ Similarly to bio-PdAu nanoparticles, core–shell structures of bio-PdRu were identified, but were a minority population.

## 7. Recovery and recyclability of bio-Pd

### 7.1 Bio-Pd from acidic waste

A promising feature of microbial bioreduction is the ability to synthesise bio-Pd catalysts from waste materials. High concentrations of precious metals such as Pd, Pt and Au, are recoverable from secondary sources. The Mackaskie group have made considerable progress in recovering precious metals from waste electrical and electronic equipment, such as printed circuit boards, and from spent industrial and automotive waste leachates.^204–206^ When first assessed for recovering Pd(ii) from acid leachate of spent automotive catalysts, cells of *D. desulfuricans* only recovered around 15% of Pd(ii) due to high chloride concentration.^82^ However, an effective way around this was to pre-seed the cells with a low Pd loading (1 wt%) before exposing them to the waste solution. The Pd(0) pre-seeded cells provided additional nucleation sites for the autocatalytic reduction of dissolved Pd species.^20,21,207–209^ Mabbett *et al.* used pre-seeded 1% Pd/*E. coli* to recover Pd from industrial waste leachates (pH = 1.5); which contained a mixture of metals including Pd, Pt, Rh, as well as very high Cl^−^ concentration from dissolving in aqua regia.^207^ The recovered Pd/*E. coli* catalysts were used for the reduction of 500 μM Cr(vi), and the reduction rate was dependent on the Cl^−^ concentration of the initial metal waste solution. When the Cl^−^ concentration was doubled the Cr(vi) reduction rate fell from of 0.030 to 0.005 mmol h^−1^ g^−1^, respectively. In comparison, 5% Pd/*E. coli* (wt/dry wt) synthesised from a non-waste Pd(ii) salt solution reduced Cr(vi) at a much faster rate of 0.158 mmol h^−1^ g^−1^. Murray *et al.* found that pre-seeding cells of *E. coli* with either Pd or Pt resulted in 65% recovery of PGMs from diluted acid leachate (pH = 1.6).^208^ On the other hand, blank cells gave negligible recovery, suggesting the metal was recovered mainly through abiotic reduction onto the cell-supported Pd(0) seeds. Bimetallic PdPt/*E. coli* nanoparticles were successfully recovered from model acidic solutions *via* the pre-seeding method. Pre-seeded 2% Pd/*E. coli* (wt/dry wt) resulted in the recovery of a 16 wt% total PdPt/*E. coli* from a model solution, with higher activity for Cr(vi) reduction than 2% Pd/*E. coli*.^20^ Likewise, a recovered 7.6/8.4 wt% PdPt/*E. coli* (wt/dry wt) bimetallic catalyst fully reduced 0.5 mM Cr(vi) in 3 hours.^21^ The recovered PdPt/*E. coli* catalyst also catalysed the hydrogenation of soybean oil, converting 91% of linoleic acid after 2 hours, compared to 100% conversion by a commercial 2% Pd/Al_2_O_3_ catalyst.

Søbjerg *et al.* produced Pd/*C. necator* from a spent Pd/C catalyst used for a hydrogenation reaction, which was dissolved in *aqua regia* and then neutralised.^24^*C. necator* recovered half of the waste Pd and Pd/*C. necator* catalysed the Heck coupling of *p*-iodoanisole with *n*-butylacrylate, furnishing a 90% isolated yield. *C. metallidurans* and *C. necator* also recovered precious metals from a mixed metal acidic leachate (pH = 1.4) containing Ag, Au, Pt, Pd, Rh, Ir, Ru, Pb, and Cu.^209^ Both pre-palladized and fresh cells were incubated with the leachate for 24 hours, and Pd was recovered from the solution at the same rate. The bio-Pd produced catalysed the Heck coupling of 4-iodoanisole and *n*-butylacrylate, in the presence of TBAB. For both species of bacteria the pre-palladized cells gave 100% conversions, whereas blank cells only achieved 50% conversion.

The harshly acidic leachates used to recover precious metals typically range from pH = 0–2.5. Neutrophilic bacteria can successfully recover the metals, however the enzymes used for bioreduction, such as hydrogenases, become denatured at low pH after only a few hours.^207,208^ Metal-reducing acidophilic extremophile microbes are an interesting alternative for the recovery of Pd and the formation of catalytic bio-Pd in one step. Okibe *et al.* showed that the Gram-negative aerobic acidophilic Fe(iii)-reducing bacteria, *Acidocella aromatica* and *Acidiphilium cryptum*, reduce Pd(ii) using formate, H_2_ or monosaccharides (glucose and fructose) as electron donors.^210^ Bio-Pd was first produced from synthetic solutions, and then from an acidic leachate of spent commercial Pd catalysts (pH = 2.5) containing 20 mg L^−1^ Pd(ii), 2240 mg L^−1^ Al^3+^ and 187 mM Cl^−^. Pd/*Ac. aromatica* from the acid leachate performed comparably to Pd/*Ac. aromatica* from the synthetic Pd(ii) solution for the catalytic reduction of Cr(vi). In addition, the two catalysts displayed similar median nanoparticle sizes of 18.1 and 19.6 nm, respectively. In a following study, *Ac. aromatica* was shown to reduce Au(iii) to Au(0) nanoparticles at pH = 2.5.^211^ The thermo-acidophilic archaeon *Sulfolobus tokodaii* was shown to reduce Pd(ii) from acidic solutions (pH = 2).^212^ The archaeon showed increased resistance to chloride concentration, successfully reducing Pd(ii) in the presence of up to 500 mM Cl^−^. The mean size of bio-Pd nanoparticles decreased from 25 nm to 8.7 nm when the Cl^−^ concentration increased from 0 to 50 mM; but above 50 mM Cl^−^ the size increased again. The smallest Pd/*S. tokodaii* nanoparticles were the most catalytically active, producing the highest specific Cr(vi) reduction rate of 2.0 mg Cr(vi)/L h^−1^ mg^−1^ Pd(0). Pd/*S. tokodaii* outperformed a Pd/C commercial catalyst, which recorded a rate of 0.5 mg Cr(vi)/L h^−1^ mg^−1^ Pd(0).

### 7.2 Recyclability of bio-Pd

Bio-Pd catalysts have been shown to possess superior recyclability in some studies compared to conventional Pd catalysts. The recyclability of Pd/*D. desulfuricans* was superior to a commercial 5% Pd/C catalyst for the Heck reaction.^26^ After 6 cycles the final conversion of 5% Pd/*D. desulfuricans* fell from 99% to 93%, its rate decreasing from 0.31 to 0.22 mmol min^−1^, whereas the final conversion of 5% Pd/C dropped from 98% to 77% and its rate fell from 0.28 to 0.0090 mmol min^−1^. Pd leaching from bio-Pd was found to be negligible over the 6 cycles. Bennett *et al.* also found that 25% Pd/*D. desulfuricans* maintained comparable selectivity for 2-pentene on its second cycle, albeit with a lower rate of reaction.^25^ Pd/*C. necator* achieved yields of 91%, 97%, 97% and 95% for successive cycles of the Suzuki coupling of *p*-iodotoluene and phenylboronic acid.^24^*G. sulfurreducens* supported biomagnetite abiotically reduced Pd(ii) to form a 5% Pd/Fe_3_O_4_/*G. sulfurreducens* (wt/dry wt) catalyst, which gave a 100% yield for the Heck coupling of iodobenzene with both ethyl acrylate and styrene, and was easily recoverable with a magnet. After four cycles the catalyst maintained >90% conversion, whereas a colloidal Pd catalyst could only be used twice.^213^ Tuo *et al.* produced a bimetallic catalyst on a biomagnetite support using *S. oneidensis*.^200^ The PdAu/Fe_3_O_4_/*S. oneidensis* nanoparticles were mostly 4–9.4 nm, and gave higher conversions and catalytic rate constants than Pd alone for the reduction of 4-nitrophenol, and seven other nitroaromatic compounds. The biomagnetite supported bimetallic particles were highly reusable, and after 8 cycles retained 87% of their initial activity.

## 8. Further processing and applications of bio-Pd

### 8.1 Processing

Microbial cells are robust and amenable to processing techniques such as immobilisation, pyrolysis, and the incorporation of nanomaterials, which may significantly alter the catalytic properties of bio-Pd and improve its ease of use. Bio-Pd can simply be added to liquid phase reactions as a suspension catalyst, for example to catalyse the reduction of various nitroaromatic compounds.^35,129,200,214^ However, long term storage of bio-Pd containing cell suspensions has been shown to result in Pd leaching from the cell.^215^ The preparation method of Mackaskie *et al.* involves washing bio-Pd with acetone before air drying, and grinding into a fine powder.^25,26,83,164,192^ Wood *et al.* observed that no Pd leaching occurred when washing in acetone, and that the dried palladized cells were more resistant to attrition under shear than suspended cell.^160^ This process has drastically improved the catalytic properties of H_2_/formate driven bio-Pd, as the acetone disrupts the outer cell membrane so the periplasmic Pd(0) particles are more accessible.^94,216^ Ultrasonication of the cells may aid in this, including with the addition of NaOH to further release nanoparticles from the cell.^118,128,163,192^ In addition, palladized cells can be freeze-dried for later use in reactions.^202,217^ Bio-Pd can be encapsulated in polymeric beads, which are advantageous over cell suspensions in terms of separating for reuse from a reaction mixture, preventing Pd leaching into solution, and immobilising in continuous flow reactors. However, encapsulated bio-Pd suffers mass transfer limitations, resulting in slower reaction rates.^127,218,219^

The pyrolysis (or carbonisation) of bio-Pd produces a carbon support material from the biomass whilst retaining dispersed Pd(0) nanoparticles with strong catalytic properties.^220^ Ng *et al.* pyrolysed a Pd/*S. oneidensis* biofilm resulting in a high surface area Pd/C nanocomposite, containing residual phosphorous and nitrogen dopants from the biofilm.^221^ The nanocomposite outperformed a commercial Pd nanopowder for the reduction of Cr(vi), and retained >90% of its Cr(vi) reduction capacity after five cycles. Carbon nanomaterials have been integrated into bio-Pd. Microbes can simultaneously reduce Pd(ii) and graphene oxide (GO) to form nanocomposites, in order to increase electrochemical conductivity and catalytic activity.^201,222–224^ A Pd/rGO/*S. oneidensis* catalyst produced a five-fold higher Cr(vi) reduction rate than Pd/*S. oneidensis*.^225^ The enhanced Cr(vi) reduction was attributed to the co-reduction of GO resulting in enhanced Pd immobilisation and cell viability.

Electrochemical catalysis is beyond the scope of this review, however effective fuel cell catalysts have been synthesised from bio-Pd, for example, to enhance the rate limiting oxygen reduction reaction in microbial fuel cells.^226,227^ Bio-Pd commonly undergoes modification *via* pyrolysis and/or the incorporation of rGO for use in fuel cells.^228–231^

Recent studies have incorporated various nanomaterials with bacteria, such as metal–organic frameworks (MOFs) and TiO_2_ nanotubes, which enhanced the adsorption and reduction of various environmental contaminants.^232–234^ Another study integrated the synthesis of a photoactive polymer onto microbial surfaces through bio-Pd catalysed Sonogashira polymerisation.^235^

### 8.2 Biocatalysis

The studies discussed so far have focused on the abiotic catalytic properties of bio-Pd nanoparticles. However, the catalytic properties of bio-Pd have been effectively combined with the enzymatic activity of microbial cells (from the field of “biocatalysis”), creating powerful whole cell catalysts capable of cascade reactions. In their seminal paper, Foulkes *et al.* produced bio-Pd in *E. coli* containing a genetically engineered monoamine oxidase to create a whole cell biocatalyst for the enantioselective deracemization of a cyclic.^217^ After five catalytic cycles, comprising of a monoamine oxidase catalysed resolution of a racemic mix of (*R*) and (*S*) 1-methyltetrahydroisoquinoline (MTQ), followed by bio-Pd conversion of the amine, resulting in an enantiomeric excess of 96%. Pd was recovered by a microbial community in a H_2_ driven membrane biofilm reactor to form bio-Pd nanoparticles.^236,237^ Aerobic denitrification by the biofilm reactor was significantly enhanced by the presence of bio-Pd, with nitrate reduction accomplished *via* bacterial respiration, and nitrite reduction *via* bacterial respiration and Pd(0) catalysis.^238^ In a following study the same system was effectively applied to the degradation of the toxic contaminant 4-chlorophenol (4-CP).^239^ Pd(0) catalysed the reductive dechlorination of 4-CP, with over 90% selectivity to cyclohexanone. Bacteria within the biofilm then utilised cyclohexanone as an electron donor to accelerate nitrate reduction. Similarly, superior aerobic denitrification was reported by *Bacillus megatarium* containing bio-Pd, which enhanced the activity of the denitrifying enzyme, amplified the electron transfer flux to nitrate, and abiotically catalysed the reduction of nitrite.^240^ Palladized cells of *P. putida* displayed enhanced aerobic degradation of phenol. Bio-Pd enhanced electron transfer along the enzymatic pathway that allows *P. putida* to use phenol as a growth substrate, resulting in enhanced proton motive force driving ATP production.^241^

## 9. Conclusions and future directions

Microbially supported Pd nanoparticle catalysts have been widely studied, yet contrasting results are often produced depending on the process conditions. This review gives a synopsis of the diffuse literature, and highlights the key criteria that must be considered in order to control nanoparticle formation and catalytic activity. Optimising the following parameters in lab systems may allow synthesis of bio-Pd that catalyses industrially relevant reactions with comparable activity and selectivity to commercial heterogeneous Pd catalysts.

Firstly, the species of microorganism used to produce bio-Pd is vital, as the Pd(ii) bioreduction capacity varies depending on the cell structure and enzymatic reduction systems available. In addition, the role of the cell as support material is an important factor that warrants further study. The choice of electron donor can dictate the enzymatic reduction system used, which affects size, crystallinity, monodispersity, and cellular location of bio-Pd. The degree of metal loading relative to the cell density gives rough control over the size of bio-Pd nanoparticles, and affects the cellular localisation of particles, in particular the extracellular distribution of Pd. The solution chemistry determines the Pd(ii) species that can interact with the cell, which undoubtedly plays a role in the structure and properties of the nanoparticles formed from Pd(ii) biosorption and bioreduction. Close attention needs to be paid to the: choice of buffer, pH, complexing ligands, and ions in solution. The impact of all of these parameters must be considered when comparing data from various studies of bio-Pd.

An important development is the biosynthesis of bimetallic nanoparticles, which offer drastically improved catalytic performance over monometallic bio-Pd. For example, bio-PdAg and bio-PdAu were proficient at catalysing the Suzuki coupling of aryl bromides, whereas bio-Pd gave little reactivity and often required the assistance of phase transfer catalysts. The changes in nanostructure of these bimetallic nanoparticles appear to be responsible for their enhanced catalytic properties, although more in-depth characterisation is required.

Microbial processes are of particular interest for the recovery of precious metals from industrial waste solutions, and the recovery of bio-Pd-based catalysts has been demonstrated. This early research has focused on acidic leachates, which are a challenging feedstock due to their high chloride concentrations, and wide array of metal contents, which often results in bio-Pd with inferior catalytic properties compared to bio-Pd from metal salt solutions. Optimising the bioreduction of acidic leachates may overcome this, creating highly active catalysts from waste solutions in a single step process. Here the presence of other metals in waste streams needs careful consideration, as they may have deleterious or positive impacts on catalytic capabilities. Bio-Pd catalysts are highly reusable, albeit generally with reduced reaction rates. The microbial cell makes a robust support material, as such cells are amenable to many processing treatments such as pyrolysis, freeze drying or encapsulation. Other nanomaterials may also be incorporated into the bioreduction process to create new nanocomposite materials with enhanced properties. In addition, bio-Pd can be integrated into more complex hybrid “biometallic” systems using the tools of systems biology to create powerful multifunctional biocatalysts. In summation, microbially supported Pd nanoparticles are still a relatively new sustainable biotechnology with promising applications in catalysis.

## Conflicts of interest

There are no conflicts to declare.

## Supplementary Material
